# Hyaluronic Acid Biomaterials for Central Nervous System Regenerative Medicine

**DOI:** 10.3390/cells9092113

**Published:** 2020-09-17

**Authors:** Gregory Jensen, Julianne L. Holloway, Sarah E. Stabenfeldt

**Affiliations:** 1 Chemical Engineering, School for Engineering of Matter, Transport and Energy, Arizona State University, Tempe, AZ 85224, USA; gmjense3@asu.edu; 2 School of Biological and Health Systems Engineering, Arizona State University, Tempe, AZ 85287, USA

**Keywords:** hyaluronic acid, regenerative medicine, tissue engineering, central nervous system, traumatic brain injury, spinal cord injury, scaffold

## Abstract

Hyaluronic acid (HA) is a primary component of the brain extracellular matrix and functions through cellular receptors to regulate cell behavior within the central nervous system (CNS). These behaviors, such as migration, proliferation, differentiation, and inflammation contribute to maintenance and homeostasis of the CNS. However, such equilibrium is disrupted following injury or disease leading to significantly altered extracellular matrix milieu and cell functions. This imbalance thereby inhibits inherent homeostatic processes that support critical tissue health and functionality in the CNS. To mitigate the damage sustained by injury/disease, HA-based tissue engineering constructs have been investigated for CNS regenerative medicine applications. HA’s effectiveness in tissue healing and regeneration is primarily attributed to its impact on cell signaling and the ease of customizing chemical and mechanical properties. This review focuses on recent findings to highlight the applications of HA-based materials in CNS regenerative medicine.

## 1. Introduction

Tissue engineering and regenerative medicine is a multidisciplinary field that combines principles from engineering and biology to treat damaged tissues within the human body; the primary goal is to facilitate the repair and regeneration of functional tissues and organs [1]. Tissue engineering approaches use a combination of cells, signaling molecules, and biomaterials to facilitate repair and regeneration. Biomaterials typically serve as a core template for regeneration and are generally designed to mimic the native extracellular matrix (ECM) by incorporating several biophysical and biochemical cues that direct cell function and behavior (18). The biophysical and biochemical properties of each tissue’s ECM are uniquely tailored to the specific needs of each tissue. As such, the mechanical properties, chemical composition, presentation of biochemical cues, biocompatibility, and other properties must be considered when designing an ideal material for a specific tissue. Both synthetic and naturally occurring polymers have been engineered to imitate elements of native ECM. Scaffolds based on synthetic polymers are highly tunable and excel at providing the necessary mechanical support [2]. In contrast, natural polymeric scaffolds include important biochemical cues that make them more biologically relevant [2,3]. The challenge for developing an ideal biomaterial is to merge these advantages and provide the damaged tissue with the appropriate mechanical properties and biochemical cues to support tissue healing and functional regeneration [4].To date, a range of biomaterials have shown promise in vitro and in pre-clinical animal models in the regeneration of meniscal fibrocartilage [5,6], articular cartilage [7,8], skin [9,10], bone [11,12], and periodontal tissue [13,14].

Advances in materials science and polymer chemistry have increased the synthetic versatility of natural materials to fabricate more robust scaffolds and address the traditional dichotomy between synthetic and natural polymers. Modifications to natural polymer systems can improve their biophysical tunability, including mechanical properties, while maintaining their biological activity. One natural polymer that has been extensively studied and modified for scaffold production is hyaluronic acid (HA). HA is a polysaccharide that is found in the ECM prominently throughout the human body [15] and is produced by a class of enzymes called hyaluronan synthases [16]. HA is linear in structure and ranges from 50 to 8000 kDa in normal fluids and tissues [17], depending on the tissue type [18]. As a component of the ECM, HA is biocompatible and commonly interacts with various cell receptors to coordinate cell communication and behavior [19,20,21]. Importantly, HA is amenable to a variety of simple chemical modifications that enable cross-linking between polymer chains and highly tunable scaffold formation [15,22]. HA may also be manufactured and processed using different methods to create hydrogels [23], microspheres [24], and composite hydrogel systems [25].

The application of regenerative medicine techniques becomes particularly important for the central nervous system (CNS) as the complexity of CNS tissue and its inflammatory response inhibits spontaneous healing, tissue regeneration, and functional recovery [26]. Neurological disorders are the leading cause of disability, and the second leading cause of death worldwide, with approximately 9 million deaths occurring each year (16.5% of total global deaths) [27]. The total number of deaths attributed to neurological damage or disease has also risen 34% since 1990 [27]. Of neurological disorders, stroke is the number one contributor to global disabilities, while spinal cord injury (SCI) and traumatic brain injury (TBI) are the sixth and seventh leading contributors, respectively [27]. Neurological disorders are characterized by extensive damage to the brain or spinal cord that often results in a permanent loss of motor skills and cognitive impairment [28,29,30]. Sustained, chronic loss of function due to neurological insults are the result of primary and secondary injury sequences. The initial injury causes cell death within the damaged region due to factors such as neuronal cell contusion, axonal shearing, and damage to blood vessels within the CNS [31]. Astrocytes, microglia, and other glial cells are then activated in response to cell death and damage [31]. Activation of these cells leads to a secondary injury response where local edema, hypoxic zones within the damaged region, chronic cytokine generation, and reactive oxygen species formation impede tissue regeneration and functional recovery [31]. One of the major challenges in the treatment of CNS injuries is attenuating this secondary injury sequence [32]. To mitigate the long-term damage caused by the secondary sequence, recent research has focused on the use of three-dimensional (3D) scaffolds to modulate the cellular microenvironment to provide favorable conditions for tissue healing and regeneration [4].

Here, we will highlight the tremendous potential of HA-based biomaterials as regenerative medicine scaffolds to promote healing, regeneration, and functional recovery of CNS tissues. The biological activity, chemistry, and manufacturing techniques of HA relevant to the CNS will be discussed in more detail in subsequent sections.

## 2. Biological Activity of HA in the CNS

**Figure 1:**
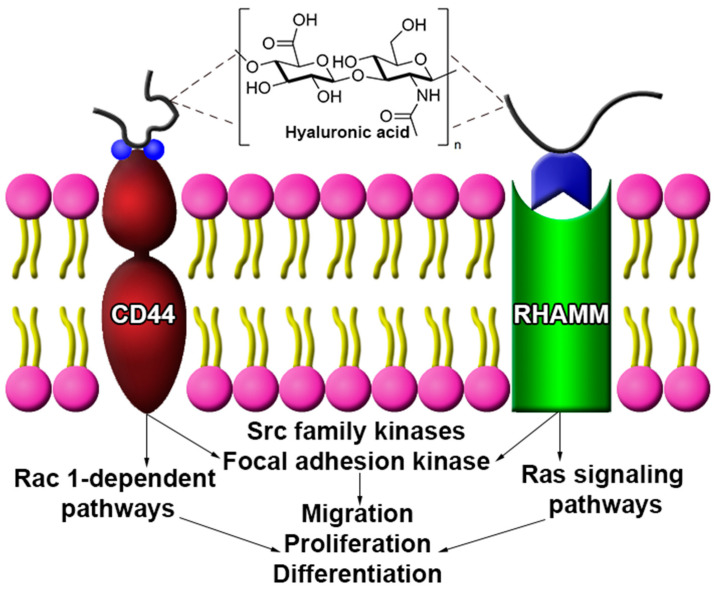
Hyaluronic acid (HA) interacts with several cell surface receptors to modulate cell behavior. While CD44 and RHAMM share common intracellular signaling pathways (i.e., Src family, focal adhesion kinase), each may act through unique pathways to elicit a specific cell behavior. CD44 engagement is linked to Rac-1-dependent signaling while RHAMM stimulates Ras-mediated signaling.

As a major component of the brain ECM, HA plays a significant role in maintaining homeostasis in neuronal tissue through influencing cell migration, proliferation, differentiation, and other cell behaviors [33,34,35]. Cell function is also influenced by the molecular weight (Mw) of HA, as low (~80 to 800 kDa) and high Mw (>1600 kDa) forms of HA elicit different cell behaviors [36,37,38,39]. One response is the production of pro-inflammatory mediators brought on by low Mw HA, and the inhibition of such mediators by high Mw HA [35,36]. HA is primarily synthesized by neurons and astrocytes, with evidence suggesting astrocytes are responsible for high Mw HA synthesis [39,40]. Localization and deposition of HA occurs around myelinated fibers and gives rise to lattice-like ECM substructures, called perineuronal nets, that surround neuronal cells and aid in developmental neuroplasticity and brain maturation [41,42,43]. HA reportedly interacts with several receptors to coordinate cell behavior; the most common HA receptors are CD44 and the receptor for hyaluronan-mediated motility (RHAMM) (Figure 1) [44].

CD44 is a cell surface receptor expressed in glial and neuronal cells, where receptor engagement with HA influences cell behavior for neural tissue under physiologic and damage/disease conditions [19,21,45,46]. For example, HA-CD44 coupling in astrocytes induced the Rac 1-dependent PKNγ (protein kinase N-γ) pathway, leading to astrocyte cytoskeletal activation and enhanced astrocyte migration under physiological conditions [47,48]. Interactions between HA and CD44 coordinate cell behaviors by activating Src family kinases (SFKs) and focal adhesion kinase (FAK) cascade [47,49]. In contrast to CD44, RHAMM is present in the cytoplasm and nucleus of cells, as well as on the cell surface [21]. RHAMM engagement with HA may activate the SFK and FAK cascade [20], and induce Ras signaling pathways to modulate cell behavior under normal physiological conditions, such as migration and proliferation [19,21].

### 2.1. Migration

In vitro assessment of astrocyte/HA interactions reveal the activation of Rac-1 signaling to modulate astrocyte migration responses of astrocytes to HA and the Rac-1 signaling pathway include enhanced migration in response to injury [48]. Bourgiugnon et al. demonstrated that astrocyte migration was enhanced by HA engagement following a scratch wound to an astrocyte monolayer culture [48]. The role of CD44 in migration was further elucidated by Piao et al. [50]. Rodent oligodendrocyte progenitor cells (OPCs) experienced increased migration when treated with HA in vitro compared to untreated controls. Furthermore, this response was inhibited via CD44 functional antibody blocking [50]. This trend was also observed in an in vivo setting where migration of OPCs transplanted within an inflamed spinal cord (zymosan-induced inflammation) was inhibited by pre-incubating OPCs with the CD44 blocking antibody [50]. Cortical ischemic lesions reportedly enhance HA expression within the effected ipsilateral hemisphere in a mouse model [51] that may serve to support HA/RHAMM mediated astrocyte migration within the peri-infarct region [51].

### 2.2. Proliferation

Proliferation of CNS cell types is also influenced by HA interactions with cell receptors. In vitro studies on astrocytes show that HA plays an important role in the formation of detrimental glial scars due to its ability to influence astrocyte proliferation [36,52]. High-Mw HA decreased astrocyte proliferation in vitro [36], while degraded high-Mw HA into low-Mw forms enhanced astrocyte proliferation in a rat SCI model [52]. Though the Mw of the HA was not specified, intracortical injection of 3% HA gels into rodents sustaining a cortical defect exhibited a decrease in astrocyte proliferation compared to control animals receiving only saline solution [53]. Neural stem cell populations are also affected by HA/CD44 interactions [45]. Rodent subgranular zone neural stem cells presenting CD44 were isolated and cultured using BrdU staining as a marker for proliferation. HA treatment to cultured neural stem cells significantly reduced BrdU incorporation compared to untreated controls indicating HA modulated proliferation [45]. Okun et al. built upon this work by demonstrating that low Mw HA reduced the proliferative capability of mouse embryonic-derived neural progenitor cells through HA ligand interactions with TLR-2 receptors in vitro [54].

### 2.3. HA Signaling in CNS Disease and Disorders

While HA can play a significant role in tissue healing and regeneration, certain CNS disease states progress by the presence of HA and its interactions with cell receptors. An experimental autoimmune encephalomyelitis (EAE) mouse model shows that HA accumulates within demyelinated lesions [39]. Accumulation of high Mw HA, and production of high Mw HA by CD44 positive astrocytes, inhibited remyelination in the spinal sections of tested mice [39]. The accumulation of high Mw HA and CD44 positive astrocytes precursor cells is also common in patients suffering from vanishing white matter disease [55]. Tissue studies from patients with vanishing white matter disease, and other white matter disorders, indicate the high levels of HA and CD44 positive cells leads to defective maturation in glial cells [39,55] through stimulation of the TLR-2 signaling pathway [56]. The TLR-4 signaling pathway is also triggered by HA stimulation and plays an important role in glioblastoma stem-like cell proliferation and differentiation [57]. During differentiation, glioblastoma stem-like cells upregulate TLR-4, release HA, and activate the TLR-4-NFκB signaling pathway [57]. Activation of this pathway causes glioblastoma stem-like cells to maintain their proliferative state, implicating HA in the tumorigenic potential of glioblastoma stem-like cells in vitro [57]. To corroborate this study, proliferation of glioblastoma multiforme cultures increased when exposed to increasing concentrations of HA, as evidenced by an increase in cultured neurosphere size [46].

## 3. Chemical Modifications of HA

HA can be easily modified with reactive functional groups to enable the formation of finely tunable 3D polymer networks. Combined with the biological relevance of HA in CNS tissues, HA is an ideal biomaterial for CNS tissue engineering and regenerative medicine applications. Modifications to HA are largely focused on three distinct functional groups: the glucuronic acid carboxylic acid, the primary and secondary hydroxyl groups, and the N-acetyl group [15]. Alterations to any of these functional groups can lead to changes in the mechanical properties, chemical properties, and biological activity of the subsequent HA biomaterial [15]. Even though it is easy to customize the properties of HA materials through chemical modifications, care must be taken to avoid over-modification as this can lead to a reduction in the biological activity of HA [58]. The degree of modification also has important implications for the mechanical properties of HA-based materials. Mechanical properties are dependent on the cross-linking density of a material, which can be altered based on the degree of modification of the HA polymer [15,59]. As HA chemistry has been extensively reviewed [15,22,60], we will selectively highlight reaction schemes of most interest for CNS tissue engineering and regenerative medicine.

Cross-linking of HA materials can be generally characterized into two main groups: covalent and noncovalent cross-linking mechanisms. Covalent cross-linking mechanisms result in materials with high stability and robust mechanical properties while noncovalent mechanisms are beneficial when a material with dynamic, reversible linkages is desired [61]. Beyond cross-linking, chemistries are also available to tether biomolecules of interest to the surface of HA. These covalent and noncovalent cross-linking mechanisms, combined with biomolecule tethering chemistries, provide the user with a toolbox to customize HA material properties for a wide range of neural tissue applications.

### 3.1. Covalent Cross-Linking Mechanisms

A common method for modifying HA is through the introduction of a thiol functional group to mediate covalent cross-linking between polymer chains [15]. Thiol functionalization is performed using carbodiimide-mediated hydrazide chemistry and targets the carboxylic acid group within HA [62]. To form a 3D polymer network, the thiol functional groups are oxidized to create covalent disulfide bonds between the polymer chains [63]. Thiol-modified HA retains HA’s innate cytocompatibility and cell viability is high at a cross-linker concentration under 2.5% (*w*/*v*) [64]. Through careful timing of the disulfide cross-linking reaction, thiol-modified HA can be injected before complete cross-linking occurs [62]. Thiol-modified HA has shown promise as a tissue-engineering scaffold in SCI in vivo models [65], neural stem cell transplantation in vivo [66], and glioblastoma in vitro models [67].

Another common HA cross-linking mechanism is the use of dihydrazide chemistry to covalently cross-link HA [15]. In this approach, HA is modified with adipic dihydrazide via the carboxylic acid group within HA using carbodiimide chemistry [22]. Dihydrazide-modified HA undergoes covalent cross-linking through the formation of hydrazone linkages with ketones and aldehydes [15]. In CNS applications, dihydrazide chemistry has been used to cross-link HA-based materials to enhance the survival and migration of neural progenitor/stem cells in vitro [23,68], study tissue reconstruction in rat models of cortical impact injury [69], and to repair tissue after controlled SCI in rodents [70].

Covalent cross-linking reactions can also form 3D HA scaffolds, where the cross-linker includes a protease degradable peptide sequence to enable cell-mediated scaffold degradation [71,72,73,74]. A protease degradable peptide cross-linker with a thiol containing cysteine residue on both ends can react with and cross-link several modifications of HA, including: thiol-modified HA [75], acrylate-modified HA [72,73], vinyl sulfone-modified HA [74], and others. As opposed to traditional hydrolytically degradable scaffolds, the degradation rate of protease degradable scaffolds is dependent on the local protease concentration. Matrix metalloproteinases (MMPs) are most commonly associated with protease degradation [76], where the protease-degradable cross-linker can be tailored to be more susceptible to certain MMPs and less sensitive to others [77]. For CNS applications, MMP-2 and MMP-9 are of interest due to higher levels of these MMPs after CNS damage [78,79,80,81]. Several MMP-cleavable peptide sequences have been reported for MMP-2 and MMP-9 [77]. Protease-degradable scaffolds are advantageous as they mimic the natural remodeling process, where MMPs are responsible for degrading the ECM during tissue remodeling and maintenance [82,83]. MMP degradable HA-based scaffolds have been utilized in SCI in rats [72], stroke in vivo models in mice [73], and 3D neuronal networks in vitro [74].

### 3.2. Noncovalent Cross-Linking Mechanisms

In contrast to covalent mechanisms that permanently cross-link HA polymer chains together, noncovalent interactions form reversible and dynamic cross-links between HA polymer chains [76]. Noncovalent bonds form through a variety of mechanisms, such as guest–host chemistry, thermal gelation, ionic interactions, hydrophobic interactions, hydrogen bonding, and protein-ligand interactions [84,85] and are responsive to a variety of stimuli (e.g., temperature, pH, shear stress, ion concentration, etc.). While HA scaffolds using noncovalent cross-linking mechanisms have not been applied extensively for CNS applications, noncovalent scaffolds display unique properties that are valuable for general regenerative medicine applications. The reversible and dynamic nature of noncovalent cross-links is advantageous for designing materials with shear-thinning and self-healing capabilities [85,86]. Such materials exhibit viscous flow at high shear strain (shear-thinning property) and recover their original mechanical properties when the strain is removed (self-healing property) [87]. Shear-thinning materials are also capable of protecting encapsulated cells from damaging shear strain during injection [88]. These materials are ideal injectable scaffolds, due to their ease of injection via shear-thinning and their rapid gelation time immediately after injection via self-healing.

A thermally responsive, shear-thinning, noncovalent scaffold can be created using a polymer blend of HA and methylcellulose (MC) [89,90]. In solution at 37 °C, MC forms a weak gel due to hydrophobic junctions which decrease with increasing temperature [91]. An increasing salt concentration reduces MC solubility causing a reduction in gelation temperature and an increase in mechanical properties [91,92]. Blending HA with MC (HAMC) has a similar effect as the anionic carboxylic acid groups within HA behave similar to salt [91]. HAMC scaffolds are not only more robust compared to MC alone, but the incorporation of HA also provides the material with shear-thinning and self-healing capabilities. The shear-thinning and self-healing properties were attributed to polymer chain entanglement; although, the exact gel recovery time was not specified [91]. In the context of the CNS, HAMC scaffolds have been used to study neural progenitor/stem cell viability and differentiation in vitro [89], as drug delivery vehicles in rodent stroke models [93,94], and to treat SCI in rodent models [95,96].

**Figure 2:**
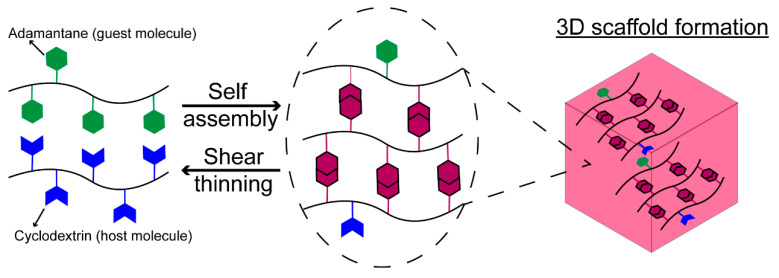
HA-based materials cross-linked using reversible guest–host chemistry. The guest and host functional chemical groups bind to form a guest–host complex, which serves as a noncovalent cross-link. The guest–host cross-links are shear-thinning and rapidly self-healing, making this platform promising for minimally invasive injection for CNS applications.

To create shear-thinning, injectable, scaffolds composed entirely of HA, guest–host chemistry can be used to form reversible, noncovalent cross-links [87,97]. In guest–host interactions, guest (e.g., adamantane, AD) and host (e.g., cyclodextrin, CD) functional groups bind to each other through hydrophobic interactions to form a guest–host complex (Figure 2). When HA is separately modified with CD (guest) and AD (host) functional groups, the guest–host complex serves as a noncovalent cross-link between the AD-modified HA and CD-modified HA polymer chains (Figure 2). The guest–host complex is reversible: de-coupling at high shear strain (i.e., injection) and rapidly reforming at low shear strain within seconds [87,97]. Compared to other HA systems, the extremely rapid self-healing behavior is advantageous in preventing scaffold diffusion into the surrounding tissue or ejection from the injection site. The mechanical properties of guest–host assembled HA hydrogels are easily tuned to match CNS tissue by altering the guest and host modification percentage within HA [98]. The combined advantages in tunability and injectability of HA-based scaffolds fabricated using guest–host chemistry opens promising avenues for this platform to be used as a tissue-engineered scaffold for CNS applications.

### 3.3. Biomolecule Tethering

While chemical modifications of HA are extremely useful, extensive HA modifications reduce the biological activity of HA by inhibiting binding to cell surface receptors [58]. To improve bioactivity or add biofunctionality, methods have been developed to covalently tether a wide range of biomolecules to HA. HA-based scaffolds have been modified with laminin (an adhesive protein common in the CNS [99]) to improve neural progenitor/stem cell migration in the direction of stromal cell-derived factor-1α gradients using in vitro and in vivo TBI models [23,68]. Schwann cells cultured on HA-based scaffolds with laminin also secreted higher levels of nerve growth factor (NGF) and brain-derived neurotrophic factor (BDNF) compared to scaffolds without laminin [100]. Further, the inclusion of laminin within HA scaffolds supported cell infiltration and angiogenesis, while inhibiting glial scar formation in a rodent SCI model [69]. As an alternative to laminin, laminin-derived peptides, IKVAV and LRE, can be tethered to HA with highly tunable control over peptide concentration without significantly impacting the original scaffold properties. Laminin-derived peptides have been used to modulate ECM degradation and enhance neurite outgrowth during in vitro studies using mouse embryonic stem cells [101] and human-induced pluripotent stem-cell-derived neural stem cells [102]. Inclusion of an RGD cell-adhesive peptide (derived from fibronectin) on HA-based scaffolds has demonstrated the ability to increase the viability of neural stem/progenitor cells in vitro [103]. Additional studies indicate neurite outgrowth is dependent on RGD concentration, with higher levels of RGD correlated with increased neuron densities in vitro [104]. Modifying HA with heparin is also promising for CNS regenerative medicine applications due to its ability to bind growth factors. Growth factor binding to heparin protects them from degradation while aiding in local accumulation of growth factors to be utilized by cells [105]. HA nanofibers with heparin were used as a growth factor delivery vehicle and resulted in increased chick dorsal root ganglia neurite extension in vitro compared to either HA or heparin alone [106].

## 4. Therapeutic Relevance of HA-Based Materials in the CNS

**Figure 3:**
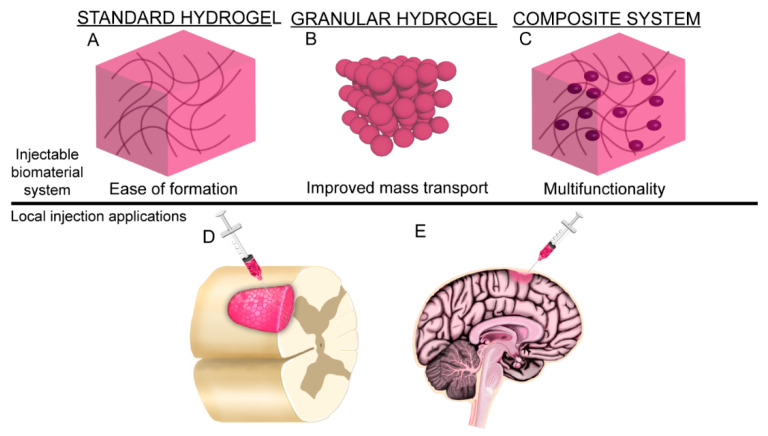
The combined versatility in HA chemical modifications and processing enables fabrication of a wide range of HA-based materials. (**A**) Hydrogels are the most common HA-based material employed in CNS applications and are beneficial due to their ease of formation. (**B**) Granular hydrogels (or microgels) offer improved mass transport through the interstitial space between particles. (**C**) Composite systems incorporate multiple phases and/or materials to add multifunctionality to the scaffold. HA-based materials can be designed for minimally invasive injection into CNS tissue to promote healing and regeneration, including (**D**) spinal cord injury and (**E**) traumatic brain injury.

The chemical modifications further diversify the processing and fabrication techniques that may be employed to generate 3D HA scaffolds. Simple modifications to processing procedures enables the formation of HA hydrogels [74], granular hydrogels (microgels) [107], electrospun fibers [106], and composite HA-based materials [96]. Different forms of HA scaffolds have unique properties that provide different benefits to CNS regenerative medicine (Figure 3).

### 4.1. Hydrogels

Hydrogels are 3D, water-swollen polymeric networks formed by chemical and/or physical interactions [18]. A major benefit for using hydrogels in tissue engineering constructs is the ease to process and form, as well as to customize the mechanical and biochemical properties to mimic soft tissues [15,59,108]. Covalently linked HA hydrogels have demonstrated the ability to support the desired growth, behavior, and function in neuron [74], Schwann [109], and neural progenitor in vitro cell cultures [68,110,111,112]. HA hydrogels are also known to modulate cell behavior. Astrocyte behavior is influenced by HA, where astrocytes are a common target for HA-based hydrogel therapies. In vivo rodent models of TBI [69] and SCI [70] show that HA hydrogels were able to recruit astrocytes to the damaged area. Levels of reactive astrocytes, which contribute to detrimental glial scar formation, were reduced in HA hydrogel treated groups compared to control animals as measured by GFAP [69,70]. The regenerative response was further augmented by an increase in angiogenesis within the injured region of the CNS [69,70]. Comparisons between gelatin hydrogels and gelatin/HA hydrogels have shown that the addition of HA into the scaffold promotes better contiguity between the scaffold and host tissue [113]. The healing potential of HA-based materials may be partially attributed to HA’s ability to encourage cellular interactions between hydrogels and CNS tissue.

HA-based hydrogels also enable the localized delivery of therapeutic cargo (e.g., stem cells, drugs, growth factors) to areas of CNS damage or disease resulting in improved therapeutic efficacy. For stem cell delivery applications, the tunability of HA-based hydrogels enables the design of a tailored microenvironment to influence stem cell fate and provide protection to transplanted cells. These attributes of HA-based hydrogels lead to enhanced transplant cell retention [23], and regulate basic cell behaviors such as migration, proliferation, and differentiation [23,114,115,116,117]. Within the brain and spinal cord, the delivery of stem cells via HA-based hydrogels has improved functional recovery in rodent SCI [116] and TBI [118] models and regeneration of nerve cells following TBI [119]. Within the spinal cord, HA hydrogels enhance stem cell survival and desired differentiation after transplantation into an in vivo SCI contusion model [116]. The results of this study indicate that the delivery of stem cells within HA hydrogels improved animal survival and Basso, Beattie, Bresnahan (BBB) Locomotor Rating scores in open-field locomotor assessments compared to either cells or HA hydrogels alone [116]. HA-based hydrogels have also shown greater utility in stem cell therapies over cells or sodium alginate hydrogels alone in rodent TBI models [118,119]. Increased stem cell viability and proliferation were observed in HA-based hydrogels over sodium alginate hydrogels before transplantation into a rodent weight drop brain injury model [119]. Once transplanted, stem cell-laden HA-based hydrogels upregulated the expression of critical neural differentiation, cell survival, and neurotrophy related genes (NSE, NEUN, MAP2, Bcl-2, BDNF, NGF) to promote stem cell differentiation, nerve cell survival, and functional recovery in rats after injury [119]. Neurotrophic growth factor secretion from transplanted cells also impacts CNS functional recovery. Neural stem cells transplanted within HA hydrogels to a rodent weight drop TBI model secreted higher levels of BDNF compared to cells transplanted without hydrogel [118]. This additional neurotrophic support was correlated with enhanced functional recovery.

Growth factors and small molecule drugs can also be directly delivered via HA-based hydrogels to elicit a desired therapeutic response. Biomolecules can be tethered to the HA polymer chain or embedded in a hydrogel for local delivery. The biophysical properties of HA hydrogels (e.g., degradation rate) can be tailored through chemical modifications to tune the drug release profile. Localized neurotropic and angiogenic growth factors, such as neurotrophin-3 (NT-3) and vascular endothelial growth factor (VEGF), improve functional outcomes after a sustained CNS injury. Elliot et al. [120] showed that HA hydrogel delivery of NT-3 improved motor function recovery in rats by promoting axonal regeneration after SCI [120]. While VEGF is more universally found throughout the body than NT-3, it still has significant impact on the CNS. In particular, delivery of a VEGF mimetic peptide effectively promoted angiogenesis and inhibited glial scar formation in a rat following removal of cortical tissue in the left frontal cortex region [32]. As glial scar formation and damaged vasculature are barriers to tissue healing and regeneration, the delivery of VEGF and its mimetic peptides have the potential to repair defects of the CNS. HA hydrogels have further shown promise as effective carriers of anti-cancer drugs in the treatment of glioblastomas (GBM) [121,122].

3D hydrogel systems, including HA, also provide a platform to model neural injury and disease. Such models enable controlled systems to elucidate fundamental mechanisms of injury/disease and evaluate novel therapeutic strategies. HA model systems illuminate the role of mechanobiology on neural progenitor fate. Wu et al. discovered that neural progenitor cell spheroid displayed greater levels of neurite outgrowth and spontaneous differentiation when cultured in softer gels compared to stiffer gels [123]. Koss et al. developed a 3D culture model based on HA hydrogels to study the inflammatory processes and glial scar formation associated with CNS insults and found that inclusion of a basal lamina mixture within the hydrogel promoted better cell integration with the matrix compared to HA alone [124]. In vitro HA-based hydrogel models of GBM tumors/cells provide important insights into the mechanisms behind GBM behavior and progression that make this type of cancer so pervasive [67,125,126].

### 4.2. Granular Hydrogels and Microgels

Granular hydrogels are a collection of hydrogel microparticles (microgels), often formed with microfluidic devices, that exhibit properties of injectability and microporosity [127,128]. A benefit of granular hydrogels is that the size of the interstitial pores between microparticles is sufficient to support cell migration and mass transport through the material [127]. Initial results with granular hydrogels for CNS applications indicate that they hold promise in modulating CNS inflammatory responses [24] and in controlled delivery of therapeutic molecules [128]. Sideris et al. used granular hydrogels to promote repair after induced stroke in mice and found that the granular hydrogel promoted astrocyte infiltration into the stroke cavity while reducing glial scar formation post-injury [107]. Related experiments in rodent stroke models also demonstrated an increase in neural progenitor cell infiltration into the stroke cavity after treatment with granular hydrogels [24]. In both studies, the application of granular hydrogels improved healing response over sham groups by improving angiogenesis and directing astrocytes and microglia into pro-repair phenotypes and behaviors [24,107], indicating the promise of this platform for CNS regeneration and repair.

### 4.3. Composite Material Systems

Composite material systems combine multiple material formations (e.g., hydrogels, microgels, fibers, etc.) into a single HA-based scaffold for tissue repair and regeneration. Composite scaffolds address the need to develop an intervention where multifunctionalities are necessary. A common example of this strategy is embedding microgels or nanoparticles inside of HA-based hydrogels. The HA hydrogel provides 3D structure while the microgels/nanoparticles can be used to encapsulate and deliver a desired therapeutic payload, with poly(lactide-co-glycolide) (PLGA) nanoparticles often being the carrier of choice. HAMC hydrogel/PLGA nanoparticle composites have been effective drug delivery vehicles for use in SCI models. Delivery of fibroblast growth factor-2 using HAMC/PLGA composites promoted greater dorsal horn blood vessel density in rats following SCI compared to HAMC alone or with blank nanoparticles [25]. The HAMC hydrogel/PLGA nanoparticle composite system has also been used in the treatment of rodents subjected to stroke injury models [129]. Again delivering BDNF, HAMC hydrogels containing PLGA nanoparticles enhanced tissue repair and functional recovery in stroke-injured rats to a greater degree than the hydrogel alone, possibly due to a greater expression of synaptophysin in BDNF treated groups [129]. It is also possible to deliver multiple growth factors via PLGA particles embedded in HA-based hydrogels. Decreased inflammation and attenuated glial scar formation was seen in a rodent SCI model when VEGF and BDNF was delivered from a HA-based hydrogel/PLGA microparticle composite system [130]. Animals receiving this treatment showed significant improvement in locomotor skills compared to either untreated animals or animals receiving the HAMC hydrogel alone [130].

Composite systems may also incorporate additional structures such as electrospun fibers for neural progenitor/stem cell delivery [89]. Inclusion of electrospun fibers synthesized from collagen reduced stem cell survival and differentiation, while electrospun fibers of poly(ε-caprolactone-co-D,L-lactide) maintained stem cell survival and differentiation similar to HAMC alone [89]. Electroconductive HA composites have also been developed by incorporating carbon nanotube or polypyrrole into HA-based hydrogels [131]. Greater neuronal differentiation of human fetal neural stem cells and human induced pluripotent stem cell-derived neural progenitor cells in 3D culture was seen in these composite hydrogels compared to hydrogels alone [131].

## 5. Future Research Perspective

Looking towards the future, recent advances in chemistry have developed promising new polymeric cross-linking strategies for HA such as dynamic cross-links. In contrast to traditional, covalent cross-links, dynamic cross-links with shear-thinning and self-healing properties are reversible and can be locally disrupted by cells to allow complex cellular functions and behaviors [85]. This local disruption of cross-links has minimal effect on the structural integrity of the bulk material [85]. Local adaptability is necessary to maintain and differentiate neural progenitor/stem cells in vitro and is important for regulating the behavior of CNS cell types [132,133]. The use of dynamic cross-links with shear-thinning and self-healing capabilities also provides HA with ideal minimally invasive injectability: with mechanical force disrupting the cross-links to enable flow and the removal of force allowing the cross-links to reform the HA material [85,87]. Neural progenitor/stem cells are frequently injected into CNS lesions to promote tissue healing and functional regeneration alongside local cells [116]. As the knowledge surrounding dynamic hydrogels expands, the application of dynamic, HA-based materials may prove useful in regulating the natural healing and inflammatory responses of CNS tissue after injury or disease.

## 6. Conclusions

HA-based materials have played an integral role in the study and treatment of CNS disorders. The biological activity of HA, as well as the ease of chemical modifications and manufacturing, has created customizable and versatile scaffolds for CNS tissue engineering and regenerative medicine. As a naturally occurring polymer, HA-based materials will continue to play a significant role in tissue regeneration and serve as personalized platforms to augment the inherent regenerative processes within the CNS.

## References

[1] O’Brien F.J., Duffy G.P. (2015). Form and function in regenerative medicine: Introduction. J. Anat..

[2] Hinderer S., Layland S.L., Schenke-Layland K. (2016). ECM and ECM-like materials—Biomaterials for applications in regenerative medicine and cancer therapy. Adv. Drug Deliv. Rev..

[3] Guo B., Ma P.X. (2014). Synthetic biodegradable functional polymers for tissue engineering: A brief review. Sci. China Chem..

[4] Jensen G., Morrill C., Huang Y. (2018). 3D tissue engineering, an emerging technique for pharmaceutical research. Acta Pharm. Sin. B.

[5] Gastel J.A., Muirhead W.R., Lifrak J.T., Fadale P.D., Hulstyn M.J., Labrador D.P. (2001). Meniscal tissue regeneration using a collagenous biomaterial derived from porcine small intestine submucosa. Arthroscopy.

[6] Murphy C.A., Costa J.B., Silva-correia J., Oliveira J.M., Reis R.L., Collins M.N. (2018). Biopolymers and polymers in the search of alternative treatments for meniscal regeneration: State of the art and future trends. Appl. Mater. Today.

[7] Ren K., He C., Xiao C., Li G., Chen X. (2015). Injectable glycopolypeptide hydrogels as biomimetic scaffolds forcartilage tissue engineering. Biomaterials.

[8] Armiento A.R., Stoddart M.J., Alini M., Eglin D. (2018). Acta Biomaterialia Biomaterials for articular cartilage tissue engineering: Learning from biology. Acta Biomater..

[9] Suganya S., Venugopal J., Ramakrishna S., Lakshmi B.S., Dev V.R.G. (2014). Naturally derived biofunctional nanofibrous scaffold for skin tissue regeneration. Int. J. Biol. Macromol..

[10] Chaudhari A., Vig K., Baganizi D., Sahu R., Dixit S., Dennis V., Singh S., Pillai S. (2016). Future Prospects for Scaffolding Methods and Biomaterials in Skin Tissue Engineering: A Review. Int. J. Mol. Sci..

[11] Pati F., Song T.H., Rijal G., Jang J., Kim S.W., Cho D.W. (2015). Ornamenting 3D printed scaffolds with cell-laid extracellular matrix for bone tissue regeneration. Biomaterials.

[12] Qu H., Fu H., Han Z., Sun Y. (2019). Biomaterials for bone tissue engineering scaffolds: A review. RSC Adv..

[13] Zhang Y., Wang Y., Shi B., Cheng X. (2007). A platelet-derived growth factor releasing chitosan/coral composite scaffold for periodontal tissue engineering. Biomaterials.

[14] Shue L., Yufeng Z., Mony U. (2012). Biomaterials for periodontal regeneration. Biomatter.

[15] Burdick J.A., Prestwich G.D. (2011). Hyaluronic acid hydrogels for biomedical applications. Adv. Mater..

[16] Gupta R.C., Lall R., Srivastava A., Sinha A. (2019). Hyaluronic acid: Molecular mechanisms and therapeutic trajectory. Front. Vet. Sci..

[17] Cowman M.K., Lee H., Schwertfeger K.L., Mccarthy J.B., Turley E.A. (2015). The content and size of hyaluronan in biological fluids and tissues. Front. Immunol..

[18] Ratner B.D., Hoffman A.S., Schoen F.J., Lemons J.E. (2013). Biomaterials Science: An Introduction to Materials in Medicine.

[19] Turley E.A., Austen L., Moore D., Hoare K. (1993). Ras-transformed cells express both CD44 and RHAMM hyaluronan receptors: Only RHAMM Is essential for hyaluronan-promoted locomotion. Exp. Cell Res..

[20] Turley E.A., Noble P.W., Bourguignon L.Y.W. (2002). Signaling properties of hyaluronan receptors. J. Biol. Chem..

[21] Casini P., Nardi I., Ori M. (2010). RHAMM mRNA expression in proliferating and migrating cells of the developing central nervous system. Gene Expr. Patterns.

[22] Collins M.N., Birkinshaw C. (2013). Hyaluronic acid based scaffolds for tissue engineering—A review. Carbohydr. Polym..

[23] Addington C.P., Dharmawaj S., Heffernan J.M., Sirianni R.W., Stabenfeldt S.E. (2017). Hyaluronic acid-laminin hydrogels increase neural stem cell transplant retention and migratory response to SDF-1α. Matrix Biol..

[24] Nih L.R., Sideris E., Carmichael S.T., Segura T. (2017). Injection of Microporous Annealing Particle (MAP) Hydrogels in the Stroke Cavity Reduces Gliosis and Inflammation and Promotes NPC Migration to the Lesion. Adv. Mater..

[25] Kang C.E., Baumann M.D., Tator C.H., Shoichet M.S. (2012). Localized and sustained delivery of fibroblast growth factor-2 from a nanoparticle-hydrogel composite for treatment of spinal cord injury. Cells Tissues Organs.

[26] Neuroregeneration—Center for Regenerative Medicine—Mayo Clinic Research. https://www.mayo.edu/research/centers-programs/center-regenerative-medicine/focus-areas/neuroregeneration.

[27] Feigin V.L., Nichols E., Alam T., Bannick M.S., Beghi E., Blake N., Culpepper W.J., Dorsey E.R., Elbaz A., Ellenbogen R.G. (2019). Global, regional, and national burden of neurological disorders, 1990–2016: A systematic analysis for the Global Burden of Disease Study 2016. Lancet Neurol..

[28] Potential Effects|Concussion|Traumatic Brain Injury|CDC Injury Center. https://www.cdc.gov/traumaticbraininjury/outcomes.html.

[29] Recovering from Stroke|cdc.gov. https://www.cdc.gov/stroke/recovery.htm.

[30] Spinal Cord Injury. https://www.who.int/news-room/fact-sheets/detail/spinal-cord-injury.

[31] Huber-Lang M., Lambris J.D., Ward P.A. (2018). Innate immune responses to trauma. Nat. Immunol..

[32] Lu J., Guan F., Cui F., Sun X., Zhao L., Wang Y., Wang X. (2019). Enhanced angiogenesis by the hyaluronic acid hydrogels immobilized with a VEGF mimetic peptide in a traumatic brain injury model in rats. Regen. Biomater..

[33] Frischknecht R., Chang K.J., Rasband M.N., Seidenbecher C.I. (2014). Neural ECM molecules in axonal and synaptic homeostatic plasticity. Prog. Brain Res..

[34] Rauti R., Renous N., Maoz B.M. (2019). Mimicking the Brain Extracellular Matrix in Vitro: A Review of Current Methodologies and Challenges. Isr. J. Chem.

[35] Carmichael S.T. (2013). Hyaluronan, neural stem cells and tissue reconstruction after acute ischemic stroke. Biomatter.

[36] Khaing Z.Z., Milman B.D., Vanscoy J.E., Seidlits S.K., Grill R.J., Schmidt C.E. (2011). High molecular weight hyaluronic acid limits astrocyte activation and scar formation after spinal cord injury. J. Neural Eng..

[37] Rayahin J.E., Buhrman J.S., Zhang Y., Koh T.J., Gemeinhart R.A. (2015). High and Low Molecular Weight Hyaluronic Acid Differentially Influence Macrophage Activation. ACS Biomater. Sci. Eng..

[38] Maharjan A.S., Pilling D., Gomer R.H. (2011). High and low molecular weight hyaluronic acid differentially regulate human fibrocyte differentiation. PLoS ONE.

[39] Back S.A., Tuohy T.M.F., Chen H., Wallingford N., Craig A., Struve J., Luo N.L., Banine F., Liu Y., Chang A. (2005). Hyaluronan accumulates in demyelinated lesions and inhibits oligodendrocyte progenitor maturation. Nat. Med..

[40] Song I., Dityatev A. (2018). Crosstalk between glia, extracellular matrix and neurons. Brain Res. Bull..

[41] Bignami A., Asher R. (1992). Some observations on the localization of hyaluronic acid in adult, newborn and embryonal rat brain. Int. J. Dev. Neurosci..

[42] Eggli P.S., Lucocq J., Ott P., Graber W., van der Zypen E. (1992). Ultrastructural localization of hyaluronan in myelin sheaths of the rat central and rat and human peripheral nervous systems using hyaluronan-binding protein-gold and link protein-gold. Neuroscience.

[43] Giamanco K.A., Matthews R.T. (2012). Deconstructing the perineuronal net: Cellular contributions and molecular composition of the neuronal extracellular matrix. Neuroscience.

[44] Wolf K.J., Kumar S. (2019). Hyaluronic Acid: Incorporating the Bio into the Material. ACS Biomater. Sci. Eng..

[45] Su W., Foster S.C., Xing R., Feistel K., Olsen R.H.J., Acevedo S.F., Raber J., Sherman L.S. (2017). CD44 Transmembrane Receptor and Hyaluronan Regulate Adult Hippocampal Neural Stem Cell Quiescence and Differentiation. J. Biol. Chem..

[46] Hartheimer J.S., Park S., Rao S.S., Kim Y. (2019). Targeting Hyaluronan Interactions for Glioblastoma Stem Cell Therapy. Cancer Microenviron..

[47] Dzwonek J., Wilczyński G.M. (2015). CD44, Molecular interactions, signaling and functions in the nervous system. Front. Cell Neurosci..

[48] Bourguignon L.Y.W., Gilad E., Peyrollier K., Brightman A., Swanson R.A. (2007). Hyaluronan-CD44 interaction stimulates Rac1 signaling and PKNγ kinase activation leading to cytoskeleton function and cell migration in astrocytes. J. Neurochem..

[49] Skupien A., Konopka A., Trzaskoma P., Labus J., Gorlewicz A., Swiech L., Babraj M., Dolezyczek H., Figiel I., Ponimaskin E. (2014). CD44 regulates dendrite morphogenesis through Src tyrosine kinase-dependent positioning of the Golgi. J. Cell Sci..

[50] Piao J.H., Wang Y., Duncan I.D. (2013). CD44 is required for the migration of transplanted oligodendrocyte progenitor cells to focal inflammatory demyelinating lesions in the spinal cord. Glia.

[51] Lindwall C., Olsson M., Osman A.M., Kuhn H.G., Curtis M.A. (2013). Selective expression of hyaluronan and receptor for hyaluronan mediated motility (Rhamm) in the adult mouse subventricular zone and rostral migratory stream and in ischemic cortex. Brain Res..

[52] Struve J., Maher P.C., Li Y., Kinney S., Fehlings M.G., Kuntz C., Sherman L.S. (2005). Disruption of the hyaluronan-based extracellular matrix in spinal cord promotes astrocyte proliferation. Glia.

[53] Lin C.-M., Lin J.-W., Chen Y.-C., Shen H.-H., Wei L., Yeh Y.-S., Chiang Y.-H., Shih R., Chiu P.-L., Hung K.-S. (2009). Hyaluronic acid inhibits the glial scar formation after brain damage with tissue loss in rats. Surg. Neurol..

[54] Okun E., Griffioen K.J., Gen Son T., Lee J.-H., Roberts N.J., Mughal M.R., Hutchison E., Cheng A., Arumugam T.V., Lathia J.D. (2010). TLR2 activation inhibits embryonic neural progenitor cell proliferation. J. Neurochem..

[55] Bugiani M., Postma N., Polder E., Dieleman N., Scheffer P.G., Sim F.J., van der Knaap M.S., Boor I. (2013). Hyaluronan accumulation and arrested oligodendrocyte progenitor maturation in vanishing white matter disease. Brain.

[56] Sloane J.A., Batt C., Ma Y., Harris Z.M., Trapp B., Vartanian T. (2010). Hyaluronan blocks oligodendrocyte progenitor maturation and remyelination through TLR2. Proc. Natl. Acad. Sci. USA.

[57] Ferrandez E., Gutierrez O., Segundo D.S., Fernandez-Luna J.L. (2018). NFκB activation in differentiating glioblastoma stem-like cells is promoted by hyaluronic acid signaling through TLR4. Sci. Rep..

[58] Kwon M.Y., Wang C., Galarraga J.H., Puré E., Han L., Burdick J.A. (2019). Influence of hyaluronic acid modification on CD44 binding towards the design of hydrogel biomaterials. Biomaterials.

[59] Vanderhooft J.L., Alcoutlabi M., Magda J.J., Prestwich G.D. (2009). Rheological properties of cross-linked hyaluronan-gelatin hydrogels for tissue engineering. Macromol. Biosci..

[60] Trombino S., Servidio C., Curcio F., Cassano R. (2019). Strategies for hyaluronic acid-based hydrogel design in drug delivery. Pharmaceutics.

[61] Fumasi F.M., Stephanopoulos N., Holloway J.L. (2020). Reversible control of biomaterial properties for dynamically tuning cell behavior. J. Appl. Polym. Sci..

[62] Shu X.Z., Liu Y., Luo Y., Roberts M.C., Prestwich G.D. (2002). Disulfide cross-linked hyaluronan hydrogels. Biomacromolecules.

[63] Prestwich G.D. (2011). Hyaluronic acid-based clinical biomaterials derived for cell and molecule delivery in regenerative medicine. J. Control. Release.

[64] Vanderhooft J.L., Mann B.K., Prestwich G.D. (2007). Synthesis and characterization of novel thiol-reactive poly(ethylene glycol) cross-linkers for extracellular-matrix-mimetic biomaterials. Biomacromolecules.

[65] Horn E.M., Beaumont M., Shu X.Z., Harvey A., Prestwich G.D., Horn K.M. (2007). Influence of cross-linked hyaluronic acid hydrogels on neurite outgrowth and recovery from spinal cord injury. J. Neurosurg. Spine.

[66] Liang Y., Walczak P., Bulte J.W.M. (2013). The survival of engrafted neural stem cells within hyaluronic acid hydrogels. Biomaterials.

[67] Rao S.S., Dejesus J., Short A.R., Otero J.J., Sarkar A., Winter J.O. (2013). Glioblastoma behaviors in three-dimensional collagen-hyaluronan composite hydrogels. ACS Appl. Mater. Interfaces.

[68] Addington C.P., Heffernan J.M., Millar-Haskell C.S., Tucker E.W., Sirianni R.W., Stabenfeldt S.E. (2015). Enhancing neural stem cell response to SDF-1α gradients through hyaluronic acid-laminin hydrogels. Biomaterials.

[69] Hou S., Xu Q., Tian W., Cui F., Cai Q., Ma J., Lee I.-S. (2005). The repair of brain lesion by implantation of hyaluronic acid hydrogels modified with laminin. J. Neurosci. Methods.

[70] Wei Y.-T., He Y., Xu C.-L., Wang Y., Liu B.-F., Wang X.-M., Sun X.-D., Cui F.-Z., Xu Q.-Y. (2010). Hyaluronic acid hydrogel modified with nogo-66 receptor antibody and poly-L-lysine to promote axon regrowth after spinal cord injury. J. Biomed. Mater. Res. Part B Appl. Biomater..

[71] Yu J., Chen F., Wang X., Dong N., Lu C., Yang G., Chen Z. (2016). Synthesis and characterization of MMP degradable and maleimide cross-linked PEG hydrogels for tissue engineering scaffolds. Polym. Degrad. Stab..

[72] Park J., Lim E., Back S., Na H., Park Y., Sun K. (2010). Nerve regeneration following spinal cord injury using matrix metalloproteinase-sensitive, hyaluronic acid-based biomimetic hydrogel scaffold containing brain-derived neurotrophic factor. J. Biomed. Mater. Res. Part A.

[73] Moshayedi P., Nih L.R., Llorente I.L., Berg A.R., Cinkornpumin J., Lowry W.E., Segura T., Carmichael S.T. (2016). Systematic optimization of an engineered hydrogel allows for selective control of human neural stem cell survival and differentiation after transplantation in the stroke brain. Biomaterials.

[74] Broguiere N., Isenmann L., Zenobi-Wong M. (2016). Novel enzymatically cross-linked hyaluronan hydrogels support the formation of 3D neuronal networks. Biomaterials.

[75] Santhanam S., Liang J., Baid R., Ravi N. (2015). Investigating Thiol-Modification on Hyaluronan via Carbodiimide Chemistry using Response Surface Methodology. J. Biomed. Mater. Res. A.

[76] Highley C.B., Prestwich G.D., Burdick J.A. (2016). Recent advances in hyaluronic acid hydrogels for biomedical applications. Curr. Opin. Biotechnol..

[77] Patterson J., Hubbell J.A. (2010). Enhanced proteolytic degradation of molecularly engineered PEG hydrogels in response to MMP-1 and MMP-2. Biomaterials.

[78] Pijet B., Stefaniuk M., Kostrzewska-Ksiezyk A., Tsilibary P.E., Tzinia A., Kaczmarek L. (2018). Elevation of MMP-9 Levels Promotes Epileptogenesis After Traumatic Brain Injury. Mol. Neurobiol..

[79] Wu M.-Y., Gao F., Yang X.-M., Qin X., Chen G.-Z., Li D., Dang B.-Q., Chen G. (2020). Matrix metalloproteinase-9 regulates the blood brain barrier via the hedgehog pathway in a rat model of traumatic brain injury. Brain Res..

[80] Rui Q., Ni H., Lin X., Zhu X., Li D., Liu H., Chen G. (2019). Astrocyte-derived fatty acid-binding protein 7 protects blood-brain barrier integrity through a caveolin-1/MMP signaling pathway following traumatic brain injury. Exp. Neurol..

[81] Shi W.-Z., Ju J.-Y., Xiao H.-J., Xue F., Wu J., Pan M.-M., Ni W.-F. (2017). Dynamics of MMP-9, MMP-2 and TIMP-1 in a rat model of brain injury combined with traumatic heterotopic ossification. Mol. Med. Rep..

[82] Löffek S., Schilling O., Franzke C.-W. (2011). Biological role of matrix metalloproteinases: A critical balance. Eur. Respir. J..

[83] Hong L.T.A., Kim Y.-M., Park H.H., Hwang D.H., Cui Y., Lee E.M., Yahn S., Lee J.K., Song S.-C., Kim B.G. (2017). An injectable hydrogel enhances tissue repair after spinal cord injury by promoting extracellular matrix remodeling. Nat. Commun..

[84] Li X., Sun Q., Li Q., Kawazoe N., Chen G. (2018). Functional hydrogels with tunable structures and properties for tissue engineering applications. Front. Chem..

[85] Wang H., Heilshorn S.C. (2015). Adaptable Hydrogel Networks with Reversible Linkages for Tissue Engineering. Adv. Mater..

[86] Guvendiren M., Lu H.D., Burdick J.A. (2012). Shear-thinning hydrogels for biomedical applications. Soft Matter.

[87] Uman S., Dhand A., Burdick J.A. (2020). Recent advances in shear-thinning and self-healing hydrogels for biomedical applications. J. Appl. Polym. Sci..

[88] Lou J., Liu F., Lindsay C.D., Chaudhuri O., Heilshorn S.C., Xia Y. (2018). Dynamic Hyaluronan Hydrogels with Temporally Modulated High Injectability and Stability Using a Biocompatible Catalyst. Adv. Mater..

[89] Hsieh A., Zahir T., Lapitsky Y., Amsden B., Wan W., Shoichet M.S. (2010). Hydrogel/electrospun fiber composites influence neural stem/progenitor cell fate. Soft Matter.

[90] Zhuo F., Liu X., Gao Q., Wang Y., Hu K., Cai Q. (2017). Injectable hyaluronan-methylcellulose composite hydrogel crosslinked by polyethylene glycol for central nervous system tissue engineering. Mater. Sci. Eng. C.

[91] Gupta D., Tator C.H., Shoichet M.S. (2006). Fast-gelling injectable blend of hyaluronan and methylcellulose for intrathecal, localized delivery to the injured spinal cord. Biomaterials.

[92] Das B., Basu A., Maji S., Dutta K., Dewan M., Adhikary A., Maiti T.K., Chattopadhyay D. (2020). Nanotailored hyaluronic acid modified methylcellulose as an injectable scaffold with enhanced physico-rheological and biological aspects. Carbohydr. Polym..

[93] Cooke M.J., Wang Y., Morshead C.M., Shoichet M.S. (2011). Controlled epi-cortical delivery of epidermal growth factor for the stimulation of endogenous neural stem cell proliferation in stroke-injured brain. Biomaterials.

[94] Caicco M.J., Cooke M.J., Wang Y., Tuladhar A., Morshead C.M., Shoichet M.S. (2013). A hydrogel composite system for sustained epi-cortical delivery of Cyclosporin A to the brain for treatment of stroke. J. Control. Release.

[95] Pakulska M.M., Ballios B.G., Shoichet M.S. (2012). Injectable hydrogels for central nervous system therapy. Biomed. Mater..

[96] Khaing Z.Z., Agrawal N.K., Park J.H., Xin S., Plumton G.C., Lee K.H., Huang Y.-J., Niemerski A.L., Schmidt C.E., Grau J.W. (2016). Localized and sustained release of brain-derived neurotrophic factor from injectable hydrogel/microparticle composites fosters spinal learning after spinal cord injury. J. Mater. Chem. B.

[97] Loebel C., Rodell C.B., Chen M.H., Burdick J.A. (2017). Shear-thinning and self-healing hydrogels as injectable therapeutics and for 3D-printing. Nat. Protoc..

[98] Rodell C.B., Kaminski A.L., Burdick J.A. (2013). Rational design of network properties in guest-host assembled and shear-thinning hyaluronic acid hydrogels. Biomacromolecules.

[99] Ji K., Tsirka S.E. (2012). Inflammation modulates expression of laminin in the central nervous system following ischemic injury. Neuroinflammation.

[100] Suri S., Schmidt C.E. (2010). Cell-laden hydrogel constructs of hyaluronic acid, collagen, and laminin for neural tissue engineering. Tissue Eng. Part A.

[101] Perera T.H., Howell S.M., Smith Callahan L.A. (2019). Manipulation of Extracellular Matrix Remodeling and Neurite Extension by Mouse Embryonic Stem Cells Using IKVAV and LRE Peptide Tethering in Hyaluronic Acid Matrices. Biomacromolecules.

[102] Perera T.H., Lu X., Smith Callahan L.A. (2020). Effect of laminin derived peptides IKVav and LRE tethered to hyaluronic acid on HiPSC derived neural stem cell morphology, attachment and neurite extension. J. Funct. Biomater..

[103] Seidlits S.K., Liang J., Bierman R.D., Sohrabi A., Karam J., Holley S.M., Cepeda C., Walthers C.M. (2019). Peptide-modified, hyaluronic acid-based hydrogels as a 3D culture platform for neural stem/progenitor cell engineering. J. Biomed. Mater. Res. Part A.

[104] Tarus D., Hamard L., Caraguel F., Wion D., Szarpak-Jankowska A., van der Sanden B., Auzély-Velty R. (2016). Design of Hyaluronic Acid Hydrogels to Promote Neurite Outgrowth in Three Dimensions. ACS Appl. Mater. Interfaces.

[105] Mammadov R., Mammadov B., Guler M.O., Tekinay A.B. (2012). Growth factor binding on heparin mimetic peptide nanofibers. Biomacromolecules.

[106] Mays E.A., Kallakuri S.S., Sundararaghavan H.G. (2020). Heparin-hyaluronic acid nanofibers for growth factor sequestration in spinal cord repair. J. Biomed. Mater. Res. Part A.

[107] Sideris E., Yu A., Chen J., Carmichael S.T., Segura T. (2019). Hyaluronic acid particle hydrogels decrease cerebral atrophy and promote pro-reparative astrocyte/axonal infiltration in the core after ischemic stroke. BioRxiv.

[108] Hlavac N., Kasper M., Schmidt C.E. (2020). Progress toward finding the perfect match: Hydrogels for treatment of central nervous system injury. Mater. Today Adv..

[109] Wang M.-D., Zhai P., Schreyer D.J., Zheng R.-S., Sun X.-D., Cui F.-Z., Chen X.-B. (2013). Novel crosslinked alginate/hyaluronic acid hydrogels for nerve tissue engineering. Front. Mater. Sci..

[110] Seidlits S.K., Khaing Z.Z., Petersen R.R., Nickels J.D., Vanscoy J.E., Shear J.B., Schmidt C.E. (2010). The effects of hyaluronic acid hydrogels with tunable mechanical properties on neural progenitor cell differentiation. Biomaterials.

[111] Nakaji-Hirabayashi T., Kato K., Iwata H. (2009). Hyaluronic acid hydrogel loaded with genetically-engineered brain-derived neurotrophic factor as a neural cell carrier. Biomaterials.

[112] Ren Y.J., Zhou Z.Y., Cui F.Z., Wang Y., Zhao J.P., Xu Q.Y. (2009). Hyaluronic acid/polylysine hydrogel as a transfer system for transplantation of neural stem cells. J. Bioact. Compat. Polym..

[113] Zhang T., Yan Y., Wang X., Xiong Z., Lin F., Wu R., Zhang R. (2007). Three-dimensional Gelatin and Gelatin/Hyaluronan Hydrogel Structures for Traumatic Brain Injury. J. Bioact. Compat. Polym..

[114] Lam J., Lowry W.E., Carmichael S.T., Segura T. (2014). Delivery of iPS-NPCs to the stroke cavity within a hyaluronic acid matrix promotes the differentiation of transplanted cells. Adv. Funct. Mater..

[115] Führmann T., Tam R.Y., Ballarin B., Coles B., Elliott Donaghue I., van der Kooy D., Nagy A., Tator C.H., Morshead C.M., Shoichet M.S. (2016). Injectable hydrogel promotes early survival of induced pluripotent stem cell-derived oligodendrocytes and attenuates longterm teratoma formation in a spinal cord injury model. Biomaterials.

[116] Zarei-Kheirabadi M., Sadrosadat H., Mohammadshirazi A., Jaberi R., Sorouri F., Khayyatan F., Kiani S. (2020). Human embryonic stem cell-derived neural stem cells encapsulated in hyaluronic acid promotes regeneration in a contusion spinal cord injured rat. Int. J. Biol. Macromol..

[117] Li X., Tzeng S.Y., Liu X., Tammia M., Cheng Y.-H., Rolfe A., Sun D., Zhang N., Green J.J., Wen X. (2016). Nanoparticle-mediated transcriptional modification enhances neuronal differentiation of human neural stem cells following transplantation in rat brain. Biomaterials.

[118] Yao M., Chen Y., Zhang J., Gao F., Ma S., Guan F. (2019). Chitosan-based thermosensitive composite hydrogel enhances the therapeutic efficacy of human umbilical cord MSC in TBI rat model. Mater. Today Chem..

[119] Zhang K., Shi Z., Zhou J., Xing Q., Ma S., Li Q., Zhang Y., Yao M., Wang X., Li Q. (2018). Potential application of an injectable hydrogel scaffold loaded with mesenchymal stem cells for treating traumatic brain injury. J. Mater. Chem. B.

[120] Elliott Donaghue I., Tator C.H., Shoichet M.S. (2016). Local Delivery of Neurotrophin-3 and Anti-NogoA Promotes Repair after Spinal Cord Injury. Tissue Eng. Part A.

[121] Barbarisi M., Iaffaioli R.V., Armenia E., Schiavo L., De Sena G., Tafuto S., Barbarisi A., Quagliariello V. (2018). Novel nanohydrogel of hyaluronic acid loaded with quercetin alone and in combination with temozolomide as new therapeutic tool, CD44 targeted based, of glioblastoma multiforme. J. Cell Physiol..

[122] Rowland M.J., Parkins C.C., McAbee J.H., Kolb A.K., Hein R., Loh X.J., Watts C., Scherman O.A. (2018). An adherent tissue-inspired hydrogel delivery vehicle utilised in primary human glioma models. Biomaterials.

[123] Wu S., Xu R., Duan B., Jiang P. (2017). Three-dimensional hyaluronic acid hydrogel-based models for in vitro human iPSC-derived NPC culture and differentiation. J. Mater. Chem. B.

[124] Koss K.M., Churchward M.A., Jeffery A.F., Mushahwar V.K., Elias A.L., Todd K.G. (2017). Improved 3D hydrogel cultures of primary glial cells for in vitro modelling of neuroinflammation. J. Vis. Exp..

[125] Ananthanarayanan B., Kim Y., Kumar S. (2011). Elucidating the mechanobiology of malignant brain tumors using a brain matrix-mimetic hyaluronic acid hydrogel platform. Biomaterials.

[126] Xiao W., Ehsanipour A., Sohrabi A., Seidlits S.K. (2018). Hyaluronic-acid based hydrogels for 3-dimensional culture of patient-derived glioblastoma cells. J. Vis. Exp..

[127] Riley L., Schirmer L., Segura T. (2019). Granular hydrogels: Emergent properties of jammed hydrogel microparticles and their applications in tissue repair and regeneration. Curr. Opin. Biotechnol..

[128] Mealy J.E., Chung J.J., Jeong H.-H., Issadore D., Lee D., Atluri P., Burdick J.A. (2018). Injectable Granular Hydrogels with Multifunctional Properties for Biomedical Applications. Adv. Mater..

[129] Obermeyer J.M., Tuladhar A., Payne S.L., Ho E., Morshead C.M., Shoichet M.S. (2019). Local Delivery of Brain-Derived Neurotrophic Factor Enables Behavioral Recovery and Tissue Repair in Stroke-Injured Rats. Tissue Eng. Part A.

[130] Wen Y., Yu S., Wu Y., Ju R., Wang H., Liu Y., Wang Y., Xu Q. (2016). Spinal cord injury repair by implantation of structured hyaluronic acid scaffold with PLGA microspheres in the rat. Cell Tissue Res..

[131] Shin J., Choi E.J., Cho J.H., Cho A.-N., Jin Y., Yang K., Song C., Cho S.-W. (2017). Three-Dimensional Electroconductive Hyaluronic Acid Hydrogels Incorporated with Carbon Nanotubes and Polypyrrole by Catechol-Mediated Dispersion Enhance Neurogenesis of Human Neural Stem Cells. Biomacromolecules.

[132] Madl C.M., LeSavage B.L., Dewi R.E., Dinh C.B., Stowers R.S., Khariton M., Lampe K.J., Nguyen D., Chaudhuri O., Enejder A. (2017). Maintenance of neural progenitor cell stemness in 3D hydrogels requires matrix remodelling. Nat. Mater..

[133] Madl C.M., LeSavage B.L., Dewi R.E., Lampe K.J., Heilshorn S.C. (2019). Matrix Remodeling Enhances the Differentiation Capacity of Neural Progenitor Cells in 3D Hydrogels. Adv. Sci..

